# Lack of detectable short-term effects of a single dose of ivermectin on the human immune system

**DOI:** 10.1186/s13071-021-04810-6

**Published:** 2021-06-05

**Authors:** Natalie E. Wilson, Barbara J. Reaves, Adrian J. Wolstenholme

**Affiliations:** 1 Department of Infectious Diseases, University of Georgia, Athens, GA 30602 USA; 2 Center for Tropical and Emerging Global Diseases, University of Georgia, Athens, GA 30602 USA; 3 Present Address: INRAE Centre Val du Loire, 37380 Nouzilly, France

**Keywords:** Ivermectin, Lymphatic filariasis, Peripheral blood mononuclear cells, Polymorphonuclear cells, Cytokine

## Abstract

**Background:**

Ivermectin is widely used in human and animal medicine to treat and prevent parasite nematode infections. It has been suggested that its mode of action requires the host immune system, as it is difficult to reproduce its clinical efficacy in vitro. We therefore studied the effects of a single dose of ivermectin (Stromectol^®^—0.15 mg/kg) on cytokine levels and immune cell gene expression in human volunteers. This dose reduces bloodstream microfilariae rapidly and for several months when given in mass drug administration programmes.

**Methods:**

Healthy volunteers with no travel history to endemic regions were given 3–4 tablets, depending on their weight, of either ivermectin or a placebo. Blood samples were drawn immediately prior to administration, 4 h and 24 h afterwards, and complete blood counts performed. Serum levels of 41 cytokines and chemokines were measured using Luminex^®^ and expression levels of 770 myeloid-cell-related genes determined using the NanoString nCounter^®^. Cytokine levels at 4 h and 24 h post-treatment were compared to the levels pre-treatment using simple *t* tests to determine if any individual results required further investigation, taking *p* =  < 0.05 as the level of significance. NanoString data were analysed on the proprietary software, nSolver™.

**Results:**

No significant differences were observed in complete blood counts or cytokine levels at either time point between people given ivermectin versus placebo. Only three genes showed a significant change in expression in peripheral blood mononuclear cells 4 h after ivermectin was given; there were no significant changes 24 h after drug administration or in polymorphonuclear cells at either time point. Leukocytes isolated from those participants given ivermectin showed no difference in their ability to kill *Brugia malayi* microfilariae in vitro.

**Conclusions:**

Overall, our data do not support a direct effect of ivermectin, when given at the dose used in current filarial elimination programmes, on the human immune system.

*Trial registration* ClinicalTrials.gov NCT03459794 Registered 9th March 2018, Retrospectively registered https://clinicaltrials.gov/ct2/show/NCT03459794?term=NCT03459794&draw=2&rank=1.

**Graphical Abstract:**

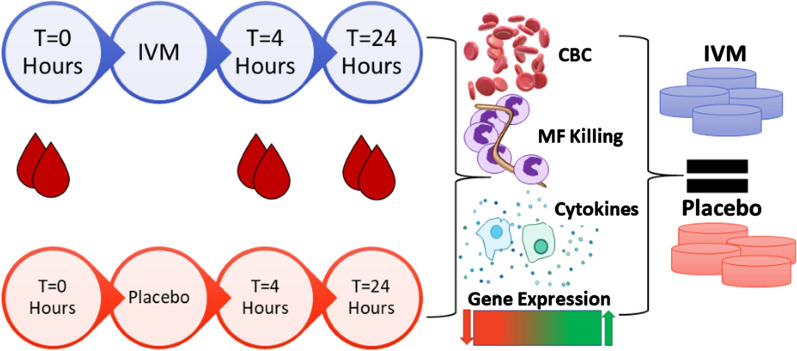

**Supplementary Information:**

The online version contains supplementary material available at 10.1186/s13071-021-04810-6.

## Introduction

Ivermectin is a key part of mass drug administration programmes for the control and eventual elimination of the major tropical diseases lymphatic filariasis (LF) and onchocerciasis, and as such is given to hundreds of millions of people every year [1–4]. Ivermectin treatment of people infected with the filarial nematodes that cause these diseases results in a rapid removal of microfilariae (Mf) from the circulation or skin of LF and onchocerciasis patients, respectively [4, 5]. The replacement of these Mf by the adult female parasites is also suppressed for many months by the drug treatment due to the embryostatic action of the drug [5], which may be due to the expression of ivermectin-sensitive glutamate-gated chloride channels in the reproductive tissues of adult females [6]. However, in vitro, concentrations of ivermectin equivalent to the peak plasma concentrations found following treatment have little or no measurable effect on Mf motility or viability [7–10]. This has led to the hypothesis that the effect of ivermectin on Mf depends in part on the host immune system [11], and some direct evidence to support this hypothesis has been obtained from studies on related animal parasites. For example, ivermectin killing of *Litomosoides carinii* (now *L. sigmodontis*) Mf in vitro was dependent on the addition of rat spleen cells [9], and this observation was later extended to neutrophils, with the ivermectin-dependent killing postulated to require nitric oxide, but not physical attachment [12]. We have previously reported that the binding of canine polymorphonuclear cells (PMNs) and peripheral blood mononuclear cells (PBMCs) to *Dirofilaria immitis* Mf in vitro increases in the presence of ivermectin [13], and this correlates with the drug-resistance status of the parasite [14]. Human PMNs and PBMCs can also bind and kill *Brugia malayi* Mf [15], though this depends on the batch of parasites used in the assay and may reflect their overall condition [16]. Taken together, these and other data could be interpreted to suggest that ivermectin has an effect not only on the parasite, but also on the host immune system [17].

The anthelmintic action of ivermectin and the other macrocyclic lactones is accepted to be due to its specific action on glutamate-gated chloride channels [18–20], at which it acts as an unconventional agonist, opening the channels slowly and effectively irreversibly [21–23]. These channels are unique to invertebrates; however, related ivermectin-sensitive channels are expressed widely in the mammalian nervous system [24] and other tissues, including on some cells of the immune system [25]. Indeed, the sensitivity of neuronal ligand-gated channels to ivermectin is the reason for the increased susceptibility of mdr-1 deficient animals to ivermectin intoxication [26]. In addition, it has more recently become apparent that ivermectin can affect multiple molecular targets in mammals, including P2X receptors and the farnesoid X receptor (FXR) [27, 28]. Ivermectin has also been shown to have anti-inflammatory properties in T cell-mediated skin disease, and this effect was independent of any effect on ligand-gated chloride channels or FXR, suggesting an additional, so far unidentified, target [29]. The drug has antiviral properties and has even been suggested as a potential therapy for COVID-19 [30, 31], though the drug concentrations at which the anti-viral effect becomes apparent are considerably higher than those found in the plasma of MDA recipients.

If the microfilaricidal action of ivermectin is due, at least in part, to the drug facilitating immune clearance of the parasites then this could be due to one of two general mechanisms, or a combination of them. There are several reports indicating that the drug can interfere with secretion and vesicle release from the parasites, which has been hypothesized to inhibit their ability to evade host immunity [32, 33], and recently these extracellular vesicles were demonstrated to downregulate the phosphorylation of mTOR in a human monocyte-derived cell line [34]. Alternatively, ivermectin could have a direct effect on the host immune system. This second hypothesis predicts that ivermectin treatment of uninfected people should result in measurable changes in immune function or in gene expression. In the experiments reported here, we have attempted to test this prediction. Such an effect could also be reflected in an increased ability of the treated immune cells to recognize and kill the parasites, and so we have also examined whether the ivermectin altered the ability of human leukocytes to kill *B. malayi* Mf in vitro.

## Materials and methods

### Ivermectin administration to human volunteers and sample collection

Twelve volunteers, aged between 18 and 65 and weighing between 50 and 84 kg, were recruited from the Athens, Georgia, area. Inclusion and exclusion criteria are listed in Additional File 1. Subjects attended the University of Georgia Clinical and Translational Research Unit (CTRU) twice. They were asked to fast for at least 3–4 h prior to the first visit. At that visit, they were weighed and allocated to groups by CTRU staff using a block randomization protocol. 18 ml of blood (Samples A) were drawn in a fasting state and subjects were administered 150 µg/kg Stromectol^®^ as three or four tablets or the equivalent number of placebo tablets immediately after blood was drawn. Participants remained at CTRU for four hours then another 15 ml of blood (Samples B) was drawn. A third blood sample (18 ml)—Samples C—was drawn 24 h after administration of the drug. All the subjects completed the study. All samples were coded immediately after being taken and all analyses completed blind. Serum was prepared from 5 to 10 ml of blood collected as above but allowed to clot in the absence of heparin or EDTA (incubated for ~ 2 h at room temperature). The liquid fraction of the blood sample was removed to a fresh tube and centrifuged at 10,000×*g* for 5 min. The resulting supernatant was filter sterilized.

### Complete blood counts

Complete blood counts (CBC) were carried out by the Clinical Pathology laboratory at Piedmont Athens Regional Hospital.

### Cytokine measurements

Human cytokine and chemokine levels were measured on a Luminex MAGPIX^®^ instrument using the Milliplex Human Cytokine/Chemokine Magnetic Bead Premixed 41 Plex Kit (EMD Millipore, Billerica MA, USA). All samples were measured using three technical replicates, with two replicates of all standards and controls.

### Gene expression analysis

PBMCs and PMNs were purified, and RNA extracted from cells isolated from each individual subject as described previously [15]. Neutrophils were isolated using the EasySep™ Direct Human Neutrophil Isolation Kit (STEMCELL Technologies, Vancouver, BC, Canada) according to manufacturer’s instructions. PBMCs were isolated using SepMate™-50 Tubes (STEMCELL Technologies, Vancouver, BC, Canada) according to manufacturer’s instructions. To remove contaminating platelets, the optional extended wash step (120×*g* for 10 min) of the SepMate™ protocol was included. Isolated PMNs and PBMCs were washed in PBS (centrifuged at 300×*g* for 5 min), re-suspended in a 1:1 mixture of RPMI-1640-10 mM HEPES buffer and autologous serum, stored at room temperature, and used within 6 h post-isolation. Cell counts were performed on a 1:10 dilution of each preparation of cells containing 0.4% trypan blue (Gibco, Life Technologies, Grand Island, NY, USA) using a BioRad TC10™ automated cell counter (BioRad, Hercules, CA, USA). To extract RNA, 0.75 ml TRIzol was added per 0.25 ml sample (5–10 × 10^6^ cells). Cells were lysed by pipetting up and down. Homogenized samples were incubated for 5 min at room temperature. Chloroform (0.2 ml/1 ml TRIzol) was added and the sample shaken for 15 s followed by a 3-min incubation at room temperature. Samples were centrifuged at 12,000×*g* for 15 min at 4 °C. The aqueous phase was removed, and the RNA precipitated by adding 0.5 ml isopropanol (including 15 µg GlycoBlue™ coprecipitant, Invitrogen, Carlsbad, CA) to the aqueous phase and incubating at room temperature for 10 min. After centrifugation at 12,000×*g* for 10 min at 4 °C, the supernatant was removed and the pellet washed in 1 ml 75% ethanol per 1 ml TRIzol. After a brief vortex, the sample was centrifuged at 7500×*g* for 5 min (4 °C). Ethanol was removed and the pellet air-dried for 5–10 min prior to resuspending in RNase-free water. The expression levels of 770 genes for each individual subject were measured on an nCounter^®^ SPRINT molecular profiling system, using the nCounter^®^ Human Innate Immunity Myeloid Panel.

### Microfilarial killing

PBMCs and PMNs were isolated from the samples A and C and incubated with freshly isolated *B. malayi* Mf as previously described [15]. Briefly, assays were set up in Corning^®^ 96-well TC-treated microplates (Millipore-Sigma, Burlington, MA, USA). Four components were added to each well in 50 µl volumes, giving a total volume of 200 µl: ~ 100 *B. malayi* Mf, autologous serum and either no cells, ~ 150,000 PMNs, or ~ 150,000 PBMCs. To create the respective controls, 50 µl of RPMI-1640 was substituted for the relevant component. The tissue culture plates were incubated at 37 °C and 5% CO_2_. Viable Mf were counted on a Nikon™ TS2 microscope (Nikon Instruments Inc., Melville, NY, USA) at 1, 24 and 120 h post-experimental set up. Mf were considered to be ‘viable’ if there was any detectable movement observed within approximately 10–20 s [8, 15].

### Statistical analysis

The mean numbers of each cell type were calculated for each arm at 24 h post-treatment and compared to those in the pre-treatment samples using a simple *t* test, with *p* =  < 0.05 as the level of significance. Since none of the measurements passed this test, no further analysis was performed. Cytokine levels were obtained from the Luminex data. The mean level plus or minus standard error of each analyte were calculated for each arm at each time point (0, 4, 24 h post-treatment). For the control and ivermectin arms, levels at 4 h and 24 h post-treatment were compared to the levels pre-treatment using simple *t* tests to determine if any individual results required further investigation, taking *p* =  < 0.05 as the level of significance. The change in levels 4 h and 24 h post-treatment were also compared between the placebo- and ivermectin-treated groups. Since none of the individual analyte results met this criterion, no further analysis was carried out. The NanoString data were analysed on the proprietary software, nSolver™ (NanoString Technologies, Inc., Seattle, WA, USA). The data passed quality control criteria. The software calculated the log_2_ geometric mean levels of each mRNA measured and used *t* tests to determine statistically significant changes in expression between 4 and 24 h post-treatment for the control and ivermectin arms. We also compared the changes in expression at 4 h and 24 h post-treatment between the placebo- and ivermectin-treated groups. The Benjamini-Yekutieli false discovery rate [35] method was used to account for the expectation that significant changes in genes may be correlated with or dependent on each other, and the subsequent resulting false discovery rate adjusted *p*-value of < 0.05 used to determine those changes that were deemed to be statistically significant. In all the analyses, each individual subject was treated as a biological replicate, giving *N* = 4 for the placebo arm and *N* = 8 for the ivermectin arm.

## Results

### Complete blood counts

**Fig. 1:**
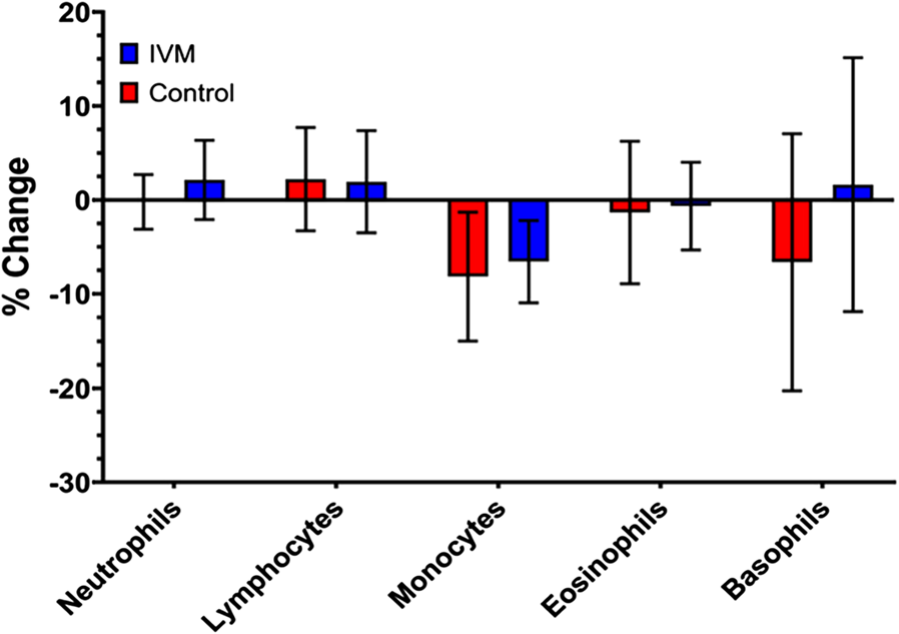
Complete blood counts show no significant changes 24 h following ivermectin administration. The mean% change in each cell type between Samples C and A is shown; error bars indicate the SEM. Ivermectin-treated subjects are in blue, placebo-treated subjects in red

After completion of all the experimental measurements, the coding was broken and we found that four of the participants received the placebo and eight received ivermectin. A comparison of the CBC results from the two groups (Fig. 1) revealed no significant differences between them 24 h after drug administration.

### Cytokine levels

**Fig. 2:**
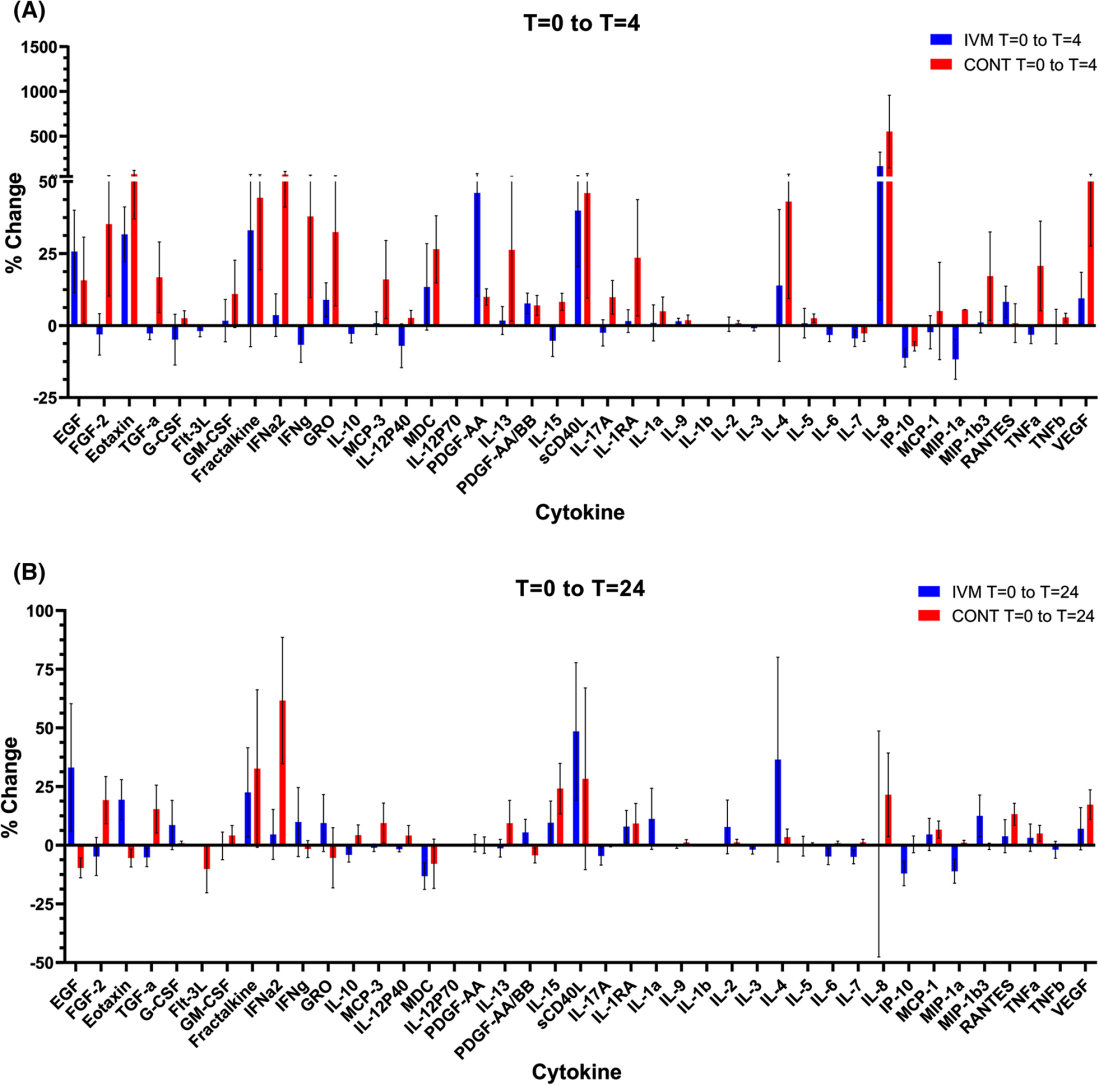
Changes in cytokine levels in ivermectin- and placebo-treated subjects at** A** 4 h and **B** 24 h post-treatment. The mean of the percentage change for each individual cytokine is shown, error bars indicate SEM. Ivermectin-treated subjects are in blue, placebo-treated subjects in red

We measured the levels of 41 cytokines and chemokines in all the samples using the Milliplex Human Cytokine/Chemokine MAGNETIC BEAD Premixed 41 Plex Kit and compared the levels at 4 and 24 h post-treatment to pre-treatment levels between the two groups (Fig. 2). This analysis was complicated by several factors. There were large individual variations in the levels of many cytokines pre-treatment. (For many analytes, at least one sample was below the limit of detection preventing a comparison of the mean levels between treatment groups, and  the detection of any changes from the baseline measurement.) Several analytes seemed to show a diurnal variation in the placebo group, as previously reported [36–39], as evidenced by changes in levels, usually increases, between *t* = 0 and *t* = 4 in the control group (Fig. 2A). Overall, there were no significant differences in the levels of any of the substances tested between the ivermectin-treated and control groups at either 4 h or 24 h post-treatment.

### Gene expression

**Fig. 3:**
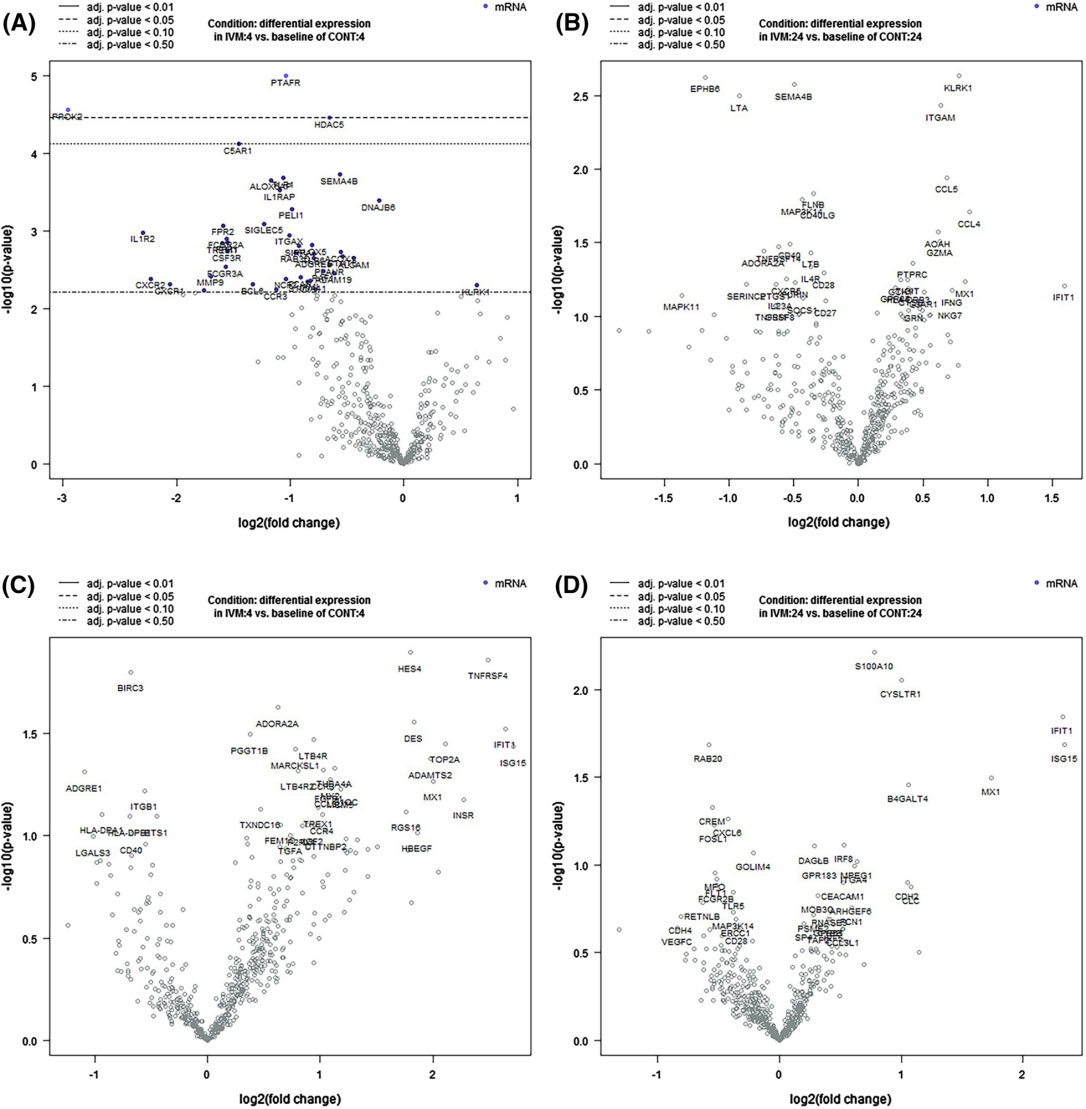
Volcano plots of changes in RNA transcript levels in PBMCs and PMNs 4 h and 24 h after administration of ivermectin or a placebo. Transcript levels were measured using the nCounter^®^ Human Innate Immunity Myeloid Panel. **A** Differential expression between ivermectin- and placebo-treated subjects in PBMCs 4 h after administration. **B** Differential expression between ivermectin- and placebo-treated subjects in PBMCs 24 h after administration. **C** Differential expression between ivermectin- and placebo-treated subjects in PMNs 4 h after administration. **D** Differential expression between ivermectin- and placebo-treated subjects in PMNs 24 h after administration

We extracted RNA from purified PMNs and PBMCs isolated from each of the experimental subjects and measured the expression of 770 transcripts in all these samples using NanoString technology and the nCounter^®^ Human Innate Immunity Myeloid Panel. The only statistically significant differences were found in the 4-h post-treatment PBMC samples (Fig. 3), where there were three transcripts that showed a reduction in expression: platelet-activating factor receptor, prokineticin 2, and histone deacetylase 5 (Table 1). There were no changes in the expression of any of these transcripts in the PMNs at either time point, or in the PBMCs at 24 h post-treatment (Fig. 3) (Additional Files 2, 3, 4, 5).

**Table 1:** PBMC transcripts with a statistically significant change in expression between ivermectin- and placebo-treated samples 4 h after administration

Transcript	Product	Log2 fold change	*p*-value	BY.*p*.value	Gene sets
PTAFR	Platelet-activating factor receptor	−1.04	1.01E−5	0.0349	Interferon signalling
PROK2	Prokineticin 2	−2.96	2.75E−5	0.0403	Angiogenesis
HDAC5	Histone deacetylase 5	−0.65	3.48E−5	0.0403	Growth factor signalling

The Benjamini-Yekutieli (BY) *p* value is that obtained after correcting for the false discovery rate [35]

### Leukocyte killing of *Brugia malayi* microfilariae

**Fig. 4:**
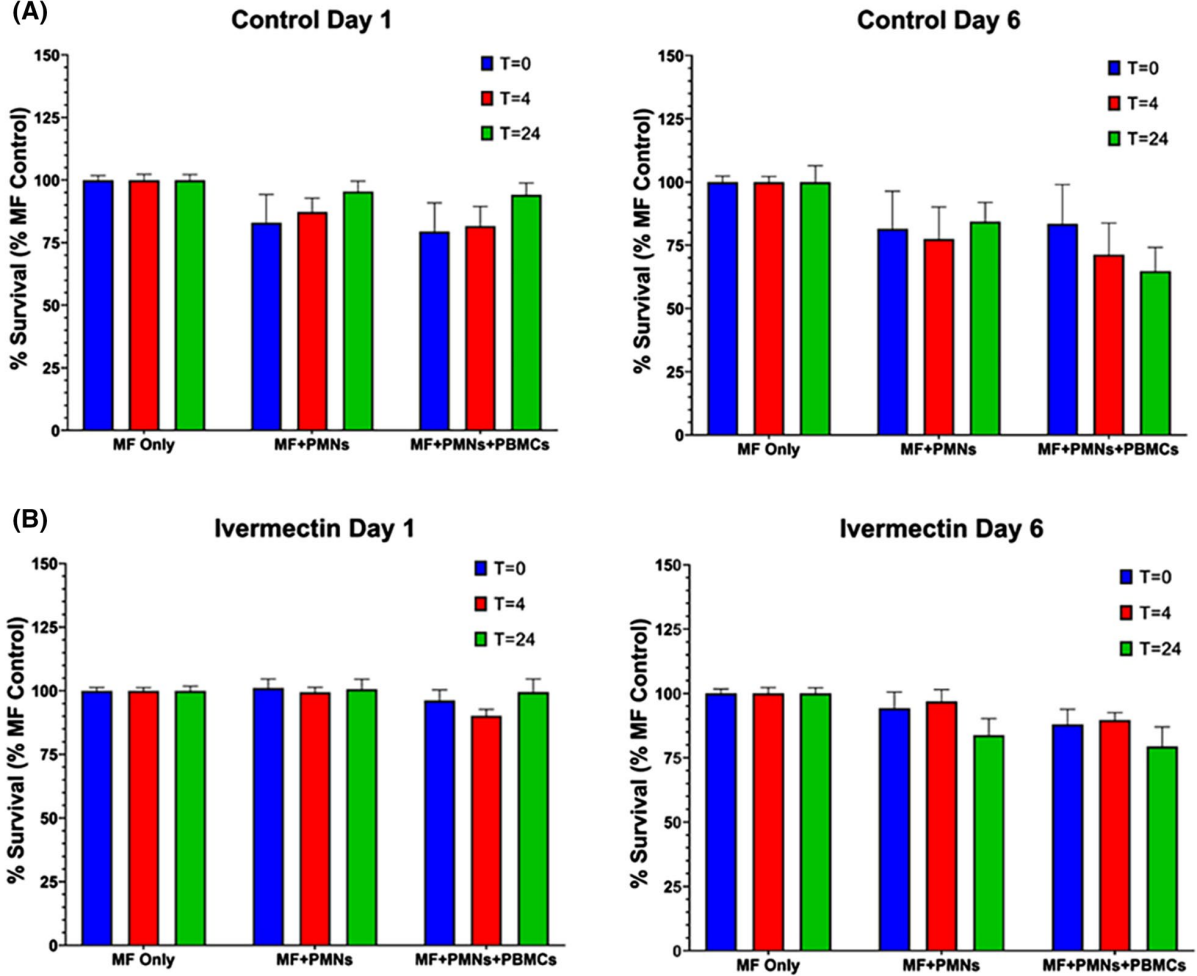
Survival of *B. malayi* Mf after incubation with PMNs or PMNs and PBMCs isolated from ivermectin- or placebo-treated subjects 4 h and 24 h after administration. **A** Survival of Mf after 1- and 6-days incubation in vitro with cells from placebo-treated subjects. **B** Survival of Mf after 1- and 6-days incubation in vitro with cells from ivermectin-treated subjects

Since we have previously shown that human PMNs and PBMCs can kill *B. malayi* Mf in vitro, we tested the effect of the ivermectin treatment on the ability of leukocytes isolated 24 h after treatment to do this (Fig. 4). We observed no reduction in the survival of Mf incubated with PMNs or PMNs + PBMCs from subjects given ivermectin, compared with those from the control subjects.

## Discussion

Ivermectin is an extremely potent and effective drug, which rapidly removes microfilaria from the circulation and tissues of infected patients. It has been speculated that this rapid clearance of microfilariae involves the host immune system [11, 17] and, in vitro, it has been shown that the drug increases interactions between immune cells and filarial parasites [12, 13]. Such an involvement might result from a direct effect of ivermectin on the mammalian immune system, or from the drug interfering with the immunomodulation produced by the parasite, or a combination of the two. Previous reports have shown that ivermectin inhibits secretion, of both proteins and extracellular vesicles, by *B. malayi* Mf [32, 33]; the experiments described here were designed to test the possibility that administration of ivermectin, at the dose given in mass drug administration programmes, has in addition a direct effect on human cellular immunity. The study was limited in size, being originally designed to provide preliminary evidence for further and more detailed studies, but we detected no evidence for any such effect. We would not have been able to detect subtle alterations in cytokine expression or gene expression but, given the extraordinary efficacy of ivermectin treatment, if this is dependent on inducing changes in the host immune response, we believe that these should have been evident in this study.

These experiments focused on PBMC and PMN as we have previously found that these cell types have the ability to interact with Mf in vitro, killing the parasites under some, but not all, circumstances [15, 16]. The complete blood counts confirmed that ivermectin did not cause any changes in the number of circulating leukocytes of any type, including eosinophils, which are frequently implicated in anti-helminth immune responses. Assessment of the effect on cytokines and chemokines was complicated by the fact that the levels of many were lower than the limits of detection of the Luminex assay, and by the variation in these levels within the control individuals. For some analytes, there seemed to be a diurnal variation, as previously reported [37–39], as the levels at 0 and 24 h were similar, whereas those at 4 h varied. However, it was not possible to distinguish any specific changes due to the drug treatment. There were similar results from the gene expression measurements. In the placebo-treated PBMC samples, we found several transcripts to be significantly changed between pre-treatment and 4 h post-treatment, but those effects disappeared after 24 h. Only three genes showed any significant variation in the drug-treated versus control individuals, and these were all in PBMC at 4 h post-treatment, suggesting that any drug effect on gene expression was transient. The largest effect was an almost eightfold reduction in prokineticin 2 mRNA expression; prokineticin 2 is a pro-inflammatory peptide that is strongly upregulated in neutrophils and other inflammatory cells in response to granulocyte-colony stimulating factor or other myeloid growth factors [40]. This downregulation might therefore lead to a short-lived anti-inflammatory response; in mice, prokineticin 2 reduces IL-10 and IL-4 production [41], so a reduction in expression in PBMCs might predict an increase in these cytokines. An increase in IL-4 was measured in ivermectin-treated individuals at 24 h but was not statistically significant. IL-10 levels did not change in either group. A prokineticin 2 antagonist also reduced interferon-γ expression in spinal lymph node cells [42], and reduced IFN-γ levels were seen in the drug-treated individuals 4 h post-administration compared to controls; again, these differences did not reach significance. The most statistically significant change was a smaller reduction, twofold, in platelet-activating factor receptor mRNA (PAFR). A role has been proposed for PAFR in inflammatory pathways [43], so this downregulation is also consistent with reduced inflammation. The third significant change was a small reduction in histone deacetylase 5 (HDAC5); this protein can also promote inflammation via activation of NF-κB [44]. Taken together, these changes are consistent with a potential anti-inflammatory effect of ivermectin [29], but the other changes in cytokine levels or gene expression that might be expected to accompany this were not detected. Also, no change in the ability of the PBMCs and PMNs isolated from treated individuals to kill *B. malayi* Mf was found. This assay uses autologous serum, so if killing were reliant on changes in compounds not tested in the Luminex assay, we should have detected it. These results suggest that any involvement of the immune system in the rapid clearance of Mf from the blood of treated individuals is due either to the inhibition of parasite-induced immunomodulation [32, 33] or to drug-induced damage to the worms that is difficult to detect or reproduce in vitro.

## Conclusions

We found no evidence to support our hypothesis that the rapid action of ivermectin against microfilaria is mediated, at least in part, via a direct effect on the host immune system. No significant changes were found in circulating cytokine levels, or in the ability of PMNs and PBMCs to kill the Mf after the drug was administered. The expression of very few genes was significantly altered.

## Supplementary Information

**Additional file 1: Table S1.** Inclusion and exclusion criteria for participants recruited into the study.

**Additional file 2: Table S2.** NanoString results for the changes in expression of the 770 genes of the myeloid gene panel in PBMCs. In this Table all the results are compared to the Control, t = 0 sample.

**Additional file 3: Table S3.** NanoString results for the changes in expression of the 770 genes of the myeloid gene panel in PMNs. In this Table all the results are compared to the Control, t = 0 sample.

**Additional file 4: Table S4.** NanoString results for the changes in expression of the 770 genes of the myeloid gene panel in PBMCs. In this Table the changes between subjects who received ivermectin and those who received a placebo are compared at 4 h and 24 h post-treatment.

**Additional file 5: Table S5.** NanoString results for the changes in expression of the 770 genes of the myeloid gene panel in PMNs. In this Table the changes between subjects who received ivermectin and those who received a placebo are compared at 4 h and 24 h post-treatment.

## Data Availability

Data supporting the conclusions of this article are included within it. The results are available at ClinicalTrials.gov under record NCT03459794.

## Abbreviations

CBC: Complete blood counts
CTRU: University of Georgia Clinical and Translational Research Unit
HDAC5: Histone deacetylase 5
LF: Lymphatic filariasis
Mf: Microfilariae
mTOR: Mechanistic target of rapamycin
PAFR: Platelet-activating factor receptor
PBMC: Peripheral blood mononuclear cells
PMN: Polymorphonuclear cells
