# Supplementary material for: Lack of detectable short-term effects of a single dose of ivermectin on the human immune system
Source: Parasit Vectors. 2021 Jun 5;14:304. doi: 10.1186/s13071-021-04810-6 (PMC8179708; doi:10.1186/s13071-021-04810-6)
Supplement: Supplementary file 1 — Additional file 1: Table S1. Inclusion and exclusion criteria for participants recruited into the study. [file 13071_2021_4810_MOESM1_ESM.pdf]

Supplementary Table S1. Inclusion and Exclusion Criteria

Inclusion Criteria	Exclusion Criteria
Weight over 50kg and under 84kg<br>Aged between 18 and 65 years	-Pregnancy or nursing mothers.<br>-Immunosuppressed individuals.<br>-Hypersensitivity to ivermectin, cellulose, starch, magnesium stearate, butylated hydroxyanisole, or citric acid powder (inert ingredients of Stromectol).<br>-Lactose intolerance (Lactose present in placebo)<br>-Recent (last 3 years) travel to West or Central Africa, or any other country where onchocerciasis is present<br>-Hepatitis/HIV<br>-Liver or renal dysfunction<br>-Currently taking any of the following medications (potential for drug interaction):<br>-- Blood thinners such as Coumadin (warfarin) or aspirin<br>-- Steroid medications (inhaled, oral or injection)<br>-- Barbiturates<br>-- Benzodiazepines such as Xanax or Klonopin<br>-- Valproic acid (Lithium)<br>-- Calcium channel blockers<br>-- Statins (cholesterol medication)
